# Pattern of Lymph Node Metastases and Recurrence in Thoracic Small Cell Esophageal Carcinoma: A Single-Institution Experience

**DOI:** 10.1245/s10434-025-18425-z

**Published:** 2025-10-07

**Authors:** Di Liu, Xiufang Lv, Jianjiao Ni, Kuaile Zhao, Jiaqing Xiang, Junhua Zhang

**Affiliations:** 1 Department of Radiation Oncology, Fudan University Shanghai Cancer Center, Shanghai, People’s Republic of China; 2 Department of Oncology, Shanghai Medical College, Fudan University, Shanghai, People’s Republic of China; 3 Shanghai Key Laboratory of Radiation Oncology, Shanghai, People’s Republic of China; 4 Department of Radiation Oncology, The First Affiliated Hospital of Zhengzhou University, Zhengzhou, Henan People’s Republic of China; 5 Department of Thoracic Surgery and State Key Laboratory of Genetic Engineering, Fudan University Shanghai Cancer Center, Shanghai, People’s Republic of China; 6 Institute of Thoracic Oncology, Fudan University, Shanghai, People’s Republic of China; 7 Department of Oncology, Shanghai Medical College, Fudan University, Shanghai, People’s Republic of China

**Keywords:** Small cell esophageal carcinoma, Lymph node metastasis, Recurrence, Prognosis

## Abstract

**Background:**

To study the patterns of lymph node metastases (LNMs) and associated factors, prognostic factors and failure patterns in primary thoracic small cell esophageal carcinoma (SCEC) after curative esophagectomy.

**Methods:**

We retrospectively reviewed thoracic SCEC patients who underwent curative esophagectomy with R0 resection at Fudan University Shanghai Cancer Center. The associations between clinicopathological variables and LNM patterns were evaluated using logistic regression. The prognostic impacts on cancer-specific survival (CSS) and disease-free survival (DFS) were assessed using Cox regression models.

**Results:**

Overall, 100/147 patients (68.0%) had LNMs (401/3560 lymph nodes, 11.3%). The frequency of LNM was 8.8% in the neck, 27.9% in the upper mediastinum (Um), 23.1% in the middle mediastinum (Mm), 15.6% in the lower mediastinum (Lm), and 35.4% in the abdomen. Patients with upper thoracic tumors (Ut) predominantly had LNMs in the Um (75.0%); patients with lower thoracic tumors (Lt) most frequently exhibited abdominal LNMs (48.0%); and patients with middle thoracic tumors (Mt) displayed a more diffuse pattern of LNMs (Mm 31.5, Um 30.3, and abdomen 28.1%). Recurrent nerve and perigastric lymph nodes had the highest metastasis/recurrence rates. Notably, lymphovascular invasion (55.1%) strongly correlated with nodal metastasis and worse DFS/CSS. Advanced stage and four or fewer chemotherapy cycles predicted poorer DFS and CSS.

**Conclusions:**

The lymphatic metastatic pattern of SCEC adheres to esophageal anatomical characteristics and tumor location. Compared with esophageal squamous cell carcinoma, SCEC displays greater lymphatic aggressiveness, with higher propensity for abdominal LNMs. Therefore, extended lymphadenectomy, particularly involving recurrent nerve and abdominal lymph nodes, combined with ≥ 4 cycles of adjuvant chemotherapy, is recommended.

**Supplementary Information:**

The online version contains supplementary material available at 10.1245/s10434-025-18425-z.

## Introduction

Esophageal carcinoma ranks among the most prevalent malignant tumors,^1^ with lymph node metastasis (LNM) representing a common metastatic pattern and a critical prognostic factor in affected patients.^2,3^ The esophagus possesses an extensive network of lymphatic capillaries that form a dense submucosal plexus; these vessels not only penetrate the esophageal wall transversely to drain into adjacent lymph nodes but also exhibit extensive longitudinal connections. Due to the non-segmental organization of this system, lymphatic flow may bypass proximal lymph nodes and disseminate to distant nodes via the plexus. As Moynihan emphasized, comprehensive knowledge of lymphatic anatomy is essential for successful surgical management of gastrointestinal malignancies, including esophageal and gastric cancers.^4^ Furthermore, investigating LNM risk factors is crucial for identifying high-risk patients and guiding personalized therapeutic strategies.

Primary small cell esophageal carcinoma (SCEC), a rare histologic subtype, accounts for only 0.5–2.8% of esophageal malignancies.^5–7^ In high-incidence regions such as East Asia, reported SCEC cases far exceed those in Western countries, representing a clinically significant patient population.^8,9^ SCEC exhibits marked aggressiveness, with over 50% of cases diagnosed at an extensive stage upon initial presentation, thereby precluding curative treatment and resulting in a dismal prognosis. Compared with esophageal squamous cell carcinoma (ESCC), SCEC demonstrates a greater propensity for early lymphatic and hematogenous spread, although robust comparative data between these histological subtypes remain scarce. Although growing evidence suggests that surgery may offer curative potential for resectable SCEC,^8–11^ its distinct LNM patterns remain poorly defined, raising questions about whether ESCC-tailored lymphadenectomy strategies are appropriate for SCEC. Moreover, SCEC patients undergoing surgery face poor outcomes, partly due to high recurrence rates. The indications for adjuvant therapy and the necessity of high-intensity chemotherapy (CT) in SCEC remain unclear, with current evidence largely derived from retrospective studies.^8,9,12–14^ These knowledge gaps underscore the need to elucidate SCEC’s pathological features and biological behavior to optimize treatment approaches. Thus, this study retrospectively analyzed primary thoracic SCEC patients who underwent curative esophagectomy at our institution, aiming to accurately define the patterns of LNM, to identify LNM-associated factors, and to systematically assess prognostic factors and failure patterns.

## Methods

### Patients

According to the practice guidelines of the Fudan University Shanghai Cancer Center (FUSCC), newly diagnosed patients underwent a complete history, physical examination, esophagogastroduodenoscopy, chest contrast-enhanced computed tomography (CT), and ultrasonography/CT of the neck and abdomen. Histopathological confirmation of SCEC was mandatory. Based on the above work-up, patients who are medically suitable, having T1–T4a tumors, with no lymph node involvement beyond the neck, thoracic cavity, and upper abdomen, and no distant metastases to other organs would undergo esophagectomy. Between January 2006 and June 2024, 147 patients with histologically confirmed SCEC underwent curative esophagectomy with R0 resection. The exclusion criteria included (1) prior neoadjuvant CT or radiotherapy; (2) mixed esophageal tumors with non-dominant small cell carcinoma components (< 50% small cell component); and (3) dissection of fewer than six lymph nodes (to ensure N-stage accuracy and surgical quality).^15^

This study received approval from the FUSCC Institutional Review Board and Independent Ethics Committee (approval number 2202251–5) and complied with the Helsinki Declaration (2013 revision). As a non-randomized retrospective analysis using de-identified data, the requirement for informed consent was waived.

### Surgical Procedure

All surgical procedures were performed according to the practice guidelines of the FUSCC.^15,16^ Our institution primarily employed three approaches: the McKeown procedure with three incisions was typically used for tumors located in the upper region, while the Ivor–Lewis procedure with two incisions and the Sweet approach with a single incision were generally used for tumors in the middle and lower regions. Nevertheless, the choice of procedure also relied on the surgeon’s preference. In the McKeown and Ivor–Lewis thoracic procedures, a muscle-sparing incision was performed in the fourth intercostal space. The azygos vein was ligated and the esophagus was mobilized from the chest apex to the diaphragm, with routine ligation of the thoracic duct above the diaphragm. During stomach mobilization, the left gastric vein and artery were closed, preserving the right gastroepiploic vessels. A 4 cm-wide gastric tube was created along the greater curvature. In the McKeown procedure, the gastric tube was routed to the left neck for manual esophagogastric anastomosis; in the Ivor–Lewis procedure, the tube was pulled into the chest for an end-to-side circular stapled anastomosis; and in the Sweet procedure, an incision was made through the sixth or seventh intercostal space, the diaphragm was cut for better access, and an esophagogastric anastomosis was performed in the left chest using a circular stapler. In this study, a gastric conduit was used for reconstruction in all patients; with the exception of 2 patients who underwent the Sweet procedure, the remaining 145 patients all underwent right thoracotomy. Patients without cervical lymph node swelling received a subtotal esophagectomy together with two-field lymphadenectomy (2-FL). In cases of enlarged lymph nodes (short diameter >5 mm) detected by cervical ultrasonography or CT, cervical dissection through a collar incision was added to make the three-field lymphadenectomy (3-FL).

### Tumor Location and Lymph Node Classification

We used the Eighth Edition American Joint Committee on Cancer (AJCC) esophageal cancer staging system to determine pathologic TNM stage and to classify thoracic SCEC in the upper, middle, and lower thoracic esophagus.^17^ The lymph node sites were classified according to the nomenclature and code numbers of the Japanese Society for Esophageal Diseases.^18^ We categorized the dissected lymph nodes into five anatomical groups: cervical, upper mediastinum (Um), middle mediastinum (Mm), lower mediastinum (Lm), and abdominal lymph nodes. Cervical lymph nodes include cervical paraesophageal lymph nodes (No. 101), deep cervical lymph nodes (No. 102), and supraclavicular lymph nodes (No. 104); Um lymph nodes include upper thoracic paraesophageal lymph nodes (No. 105), recurrent nerve lymph nodes (No. 106rec), and tracheobronchial lymph nodes (No. 106tb); Mm lymph nodes include subcarinal lymph nodes (No. 107), middle thoracic paraesophageal lymph nodes (No. 108) and main bronchus lymph nodes (No. 109); Lm lymph nodes include lower thoracic paraesophageal lymph nodes (No. 110), supradiaphragmatic lymph nodes (No. 111) and posterior mediastinal lymph nodes (No. 112); and the upper abdominal lymph nodes include perigastric lymph nodes (Nos. 1–3 and 7), common hepatic artery lymph nodes (No. 8), celiac artery lymph nodes (No. 9), and splenic artery lymph nodes (No. 11).

Patterns of LNMs in SCEC included proximal LNMs and distal LNMs according to the lymph nodes longitudinal position relative to the location of the primary tumor. Proximal LNM was defined as the nodal group at the same cross-section as the primary tumor and its nearest neighborhood groups; the other pattern was distal LNM. Nodal skip metastasis (NSM) is defined as metastatic involvement of distal lymph nodes with proximal lymph nodes free of tumor infiltration.^19^

### Endpoints Definition and Follow-Up

Baseline clinicopathological features of SCEC patients comprised demographic data, lifestyle factors (tobacco use, alcohol consumption), primary tumor location and length, histopathological features (pure or mixed SCEC subtypes, lymphovascular invasion [LVI], perineural invasion [PNI]), and adjuvant treatment. The routine examination during follow-up included physical, blood, and imaging examinations. Symptomatic patients underwent immediate diagnostic evaluation to confirm disease status. Disease recurrence/progression was categorized as local (esophagus and anastomotic), regional (regional lymph nodes), or distant (distant lymph nodes or organs, including supraclavicular lymph nodes). The last follow-up was performed on 31 December 2024, or upon patient death. Patients were followed up at the institution or by phone. Cancer-specific survival (CSS) was calculated from the date of treatment to the date of death due to tumor-related causes. Disease-free survival (DFS) was defined as the time from the start of treatment to the date of recurrence, metastasis, or death, whichever occurred first.

### Statistics

All data from this study were entered and analyzed using SPSS 26.0 (IBM Corporation, Armonk, NY, USA). Categorical variables were summarized using frequencies and percentages. Continuous data were compared using Student’s t-test, and categorical variables were analyzed using the Chi-square test or Fisher’s exact test. For those variables that demonstrated a significant association with LNMs, we employed both univariate and multivariate logistic regression models utilizing a forward stepwise selection procedure to identify significant risk factors for LNMs. Survival analysis was performed using the Kaplan–Meier method, and survival rates were compared via the log-rank test, with 95% confidence intervals (CIs) reported. The relationships between survival and clinicopathological variables were determined by Cox proportional hazards regression by calculating the hazard ratio (HR) and 95% CI in multivariate analyses. A two-sided probability value of < 0.05 was considered statistically significant.

## Results

### Patients and Clinicopathologic Features

The detailed baseline clinicopathological characteristics of 147 SCEC patients are summarized in Table 1. Most patients were male (72.1%), aged ≤ 65 years (61.2%), characterized by pure SCEC histology (78.2%), and had a history of smoking (53.7%). Tumors predominantly measured ≤ 5 cm in length (88.4%) and were located in the middle–lower thoracic esophagus (Mt: 60.5%, Lt: 34.0%). Based on the 8th edition of the TNM staging system, the majority of tumors were classified as T1b (33.3%) or T2 (36.7%). No cases of SCEC in situ were observed and only 2 patients (1.4%) had T1a-stage disease. Among the 100 patients (68.0%) with LNMs, N1 was the most common category (38.8% of these metastatic cases). Distant metastases (M1 stage) involved No. 102 and No. 104 lymph nodes in 8 affected patients. Overall, stage III disease (37.4%) represented the largest subgroup, followed by stage II (32.7%). Notably, LVI was identified in 55.1% of SCEC patients, with its prevalence increasing significantly with higher T/N stages and greater tumor length (electronic supplementary material [ESM] Table 1). Nine patients had a history of secondary tumors, all of which were treated and cured localized lesions. Regarding postoperative therapy, 118 patients (80.3%) received adjuvant CT (ACT), with 23 receiving combined radiotherapy.

**Table 1:** Clinicopathological characteristics of 147 SCEC patients

Characteristics	*N*	%
*Sex*		
Male	106	72.1
Female	41	27.9
*Age, years*		
≤ 65	90	61.2
>65	57	38.8
*Performance status*		
0	33	22.4
≥ 1	114	77.6
*Comorbidity*		
Yes	64	43.5
No	83	56.5
*Family history*		
Yes	42	28.6
No	105	71.4
*Length of disease, cm*		
≤ 3	66	44.9
3–5	64	43.5
>5	17	11.6
*Primary site*		
Ut	8	5.4
Mt	89	60.5
Lt	50	34.0
*Smoking history*		
Yes	79	53.7
No	68	46.3
*Heavy alcohol consumption*		
Yes	53	36.1
No	94	63.9
*Second tumor*		
Yes	9	6.1
No	138	93.9
*BMI, kg/m*^*2*^		
< 18.5	9	6.1
18.5–25	111	75.5
>25	27	18.4
*Pathology*		
Pure SCEC	115	78.2
Mixed ESCC/EAC	30	20.4
Mixed large cell NEC	2	1.4
*LVI*		
Yes	81	55.1
No	66	44.9
*PNI*		
Yes	29	19.7
No	118	80.3
*Surgical type*		
Sweet	2	1.4
Ivor–Lewis	101	68.7
McKeown	44	29.9
*Lymphadenectomy*		
3-FL	24	16.3
2-FL	123	83.7
*Adjuvant treatment*		
Yes	118	73.5
No	29	26.5
*T stage*		
T1a	2	1.4
T1b	49	33.3
T2	54	36.7
T3	37	25.2
T4a	5	3.4
*N stage*		
N0	47	32.0
N1	57	38.8
N2	26	17.7
N3	17	11.6
*M stage*		
M0	139	94.6
M1 (No. 102, No. 104)	8	5.4
*TNM stage*		
IB	23	15.6
IIA	19	12.9
IIB	29	19.8
IIIA	19	12.9
IIIB	36	24.5
IVA	13	8.8
IVB	8	5.4

*BMI* body mass index, *ESCC* esophageal squamous cell carcinoma, *EAC* esophageal adenocarcinoma, *NEC* neuroendocrine carcinoma, *Ut* upper thoracic, *Mt* middle thoracic, *Lt* lower thoracic, *LVI* lymphovascular invasion, *PNI* perineural invasion, *SCEC* small cell esophageal carcinoma, *2-FL* two-field lymphadenectomy, *3-FL* three-field lymphadenectomy

### Pattern of Lymph Node Metastases and Risk Factors

**Fig. 1:**
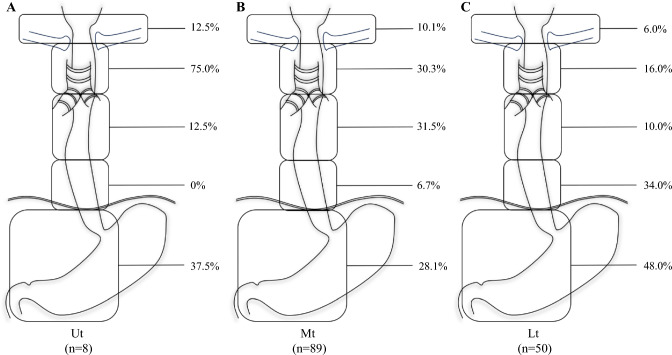
Rate of LNMs to different regions according to the location of the primary tumor. (**a**) Upper thoracic; (**b**) middle thoracic; (**c**) lower thoracic. *Ut* upper thoracic tumor, *Mt* middle thoracic tumor, *Lt* lower thoracic tumor, *LNMs* lymph node metastases

**Fig. 2:**
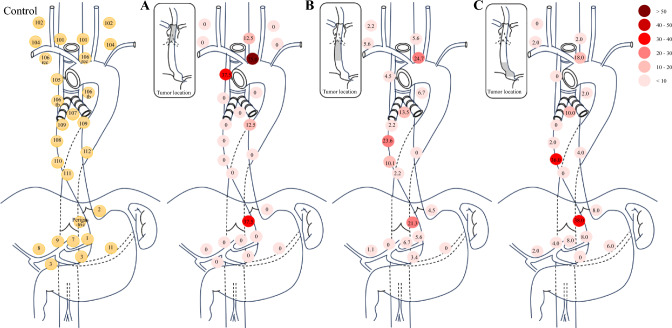
Anatomic sites of LNMs based on definitions region from the JES (8th) according to the location of the primary tumor. (**a**) Upper thoracic; (**b**) middle thoracic; (**c**) lower thoracic. *LNMs* lymph node metastases, *JES* Japan Esophageal Society

A total of 3560 lymph nodes were dissected, with 401 (11.3%) confirmed as metastatic. Among the 100 patients with LNMs, 59 patients exhibited proximal LNMs. The frequency of LNM was 8.8% in the neck, 27.9% in the Um, 23.1% in the Mm, 15.6% in the Lm, and 35.4% in the abdomen. The rates of LNMs in different locations and anatomic sites of thoracic SCEC are shown in Fig. 1 and Fig. 2. The rates of LNMs for tumors located in the upper thoracic esophagus (Ut), Mt, and Lt were 75.0, 65.2, and 70.8%, respectively. Patients with Ut tumors predominantly had LNMs in the Um (75.0%). Patients with Lt tumors most frequently exhibited abdominal LNMs (48.0%), while those with Mt tumors displayed a more diffuse pattern of LNMs (Mm 31.5%, Um 30.3%, and abdomen 28.1%). Recurrent nerve (Ut 75.0%, Mt 24.7%, Lt 18.0%) and perigastric lymph nodes (Ut 37.5%, Mt 21.3%, Lt 38.0%) had the highest metastasis rates. NSMs were observed in 26 patients (14 Mt, 12 Lt; 6 patients had undergone 3-FL and 20 had undergone 2-FL), with only 1 patient involving the cervical area, 24 patients involving the abdominal cavities, and 1 patient showing metastases in both cervical and abdominal areas.

Multivariate logistic regression analysis confirmed that only LVI remained significantly correlated with LNMs, as opposed to three associated factors (deeper tumor invasion, LVI, and greater tumor length) identified in univariate analysis (Table 2). Due to the substantial influence of LVI on LNM risk and its potential to confound other contributing factors, we examined correlations between T stage/tumor length and LVI. This analysis confirmed significant correlations of both T stage and tumor length with LVI (ESM Table 2). Moreover, only LVI showed a significant association with NSM (odds ratio 0.387, 95% CI 0.152–0.988; *p* = 0.047).

**Table 2:** Univariate and multivariate analysis of factors associated with LNM

	Variables	OR	95% CI	*p-*value
Univariate	Length of disease, cm			0.033*
	≤ 3 versus > 5	0.181	0.038–0.856	0.031*
	3–5 versus > 5	0.369	0.076–1.783	0.215
	T stage (T1–2 vs. T3–4)	0.325	0.132–0.801	0.015*
	LVI (no vs. yes)	0.131	0.059–0.291	< 0.001*
Multivariate	LVI (no vs. yes)	0.131	0.059–0.291	< 0.001*

*LNM* lymph node metastasis, *OR* odds ratio, *CI* confidence interval, *LVI* lymphovascular invasion

### Postoperative Recurrence and Lymph Node Metastasis Patterns

At the last follow-up, 38 patients remained disease-free, 109 patients experienced relapse, and 17 patients had unknown failure cause. Table 3 summarizes the recurrence patterns after the first-line treatment. Distant metastasis was the predominant failure pattern in SCEC patients (71 patients, 77.2%), with liver metastases most frequent (29 patients), followed by lung (14 patients) and bone (12 patients) [ESM Table 3]. Notably, no significant differences in recurrence patterns were observed between ACT and non-ACT groups or between ≥ 4 and < 4 ACT cycles. Distant metastasis remained the predominant failure pattern (ACT vs. no ACT: 72.2 vs. 75.7%; ≥ 4 cycles vs. < 4 cycles: 80.5 vs. 74.5%); however, ≥ 4 ACT cycles were associated with a significantly higher non-recurrence rate (12.5 vs. 38.7%; *p* = 0.001)

**Table 3:** Failure pattern after first-line treatment in SCEC patients

Failure pattern	*N*	%
*Locoregional only*	21	22.8
Local only	3	3.2
Regional only	17	18.5
Both	1	1.1
Distant only	43	46.8
*Both*	28	30.4
Regional + distant	20	21.7
Locoregional + distant	8	8.7

*SCEC* small cell esophageal carcinoma

Locoregional recurrence accounted for 22.8% of failures and 41 patients (44.6%) had regional LNMs across patterns, with metastatic lymph nodes mapped in 29 patients (70.7%): No. 106rec lymph nodes represented as the predominant metastatic site (21 patients, 72.4%), followed by abdominal lymph nodes (10 patients, 34.5%; No. 1–3 in 7 patients, No. 7–9 in 3 patients), and No. 112 lymph nodes (4 patients, 13.8%).

### Prognostic Factors for Disease-Free Survival and Cancer-Specific Survival

By 31 December 2024, 84 patients had died from cancer-related causes; the median follow-up time was 76.0 months (95% CI 44.3–107.7). The median CSS was 25.0 months (95% CI 17.1–32.9), with 1-, 3-, and 5-year CSS rates of 82.8, 39.3, and 32.7%, respectively. The median DFS for the entire cohort was 12.3 (95% CI 10.5–14.1) months, with 1- and 3-year DFS rates of 50.4 and 20.0%, respectively.

**Fig. 3:**
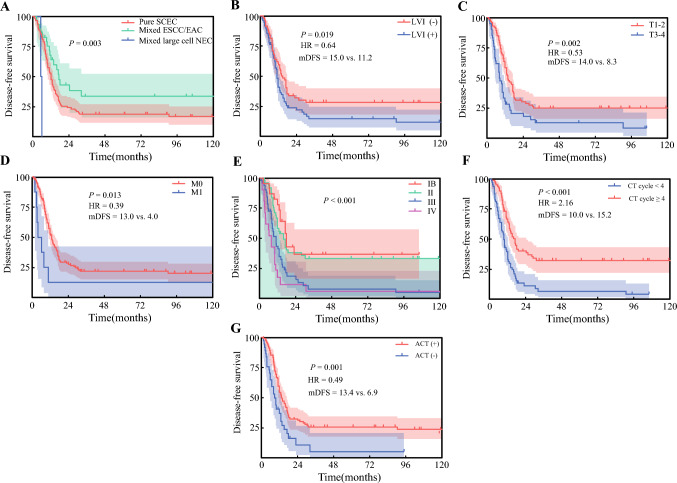
DFS curves stratified by (**a**) histology; (**b**) LVI; (**c**) T stage; (**d**) M stage; (**e**) TNM stage; (**f**) CT cycle; and (**g**) ACT in SCEC patients. *DFS* disease-free survival, *LVI* lymphovascular invasion, *TNM* tumor lymph node metastasis, *CT* chemotherapy, *ACT* adjuvant chemotherapy, *SCEC* small cell esophageal carcinoma, *ESCC* esophageal squamous cell carcinoma, *EAC* esophageal adenocarcinoma, *NEC* neuroendocrine carcinoma, *HR* hazard ratio, *mDFS* median disease-free survival

The results of the multivariate Cox proportional hazards analysis of DFS and CSS are shown in Table 4. Univariate analyses identified LVI, histologic subtype, tumor length, T stage, N stage, M stage, TNM stage, CT cycle, and ACT as significant predictors of DFS (ESM Table 4). Multivariate Cox analysis confirmed T stage, M stage, TNM stage, histologic subtype, LVI, and CT cycle as independent prognostic factors for DFS (Fig. 3).

**Table 4:** Multivariate analysis of the prognostic factors for CSS and DFS in SCEC patients

Variables	CSS		DFS	
	HR (95% CI)	*p-*value	HR (95% CI)	*p-*value
*Pathology*		–		0.002*
Pure SCEC versus mixed large cell NEC	–	–	0.16 (0.04–0.70)	0.015*
Mixed ESCC/ADC versus mixed large cell *NEC*	–	–	0.12 (0.03–0.53)	0.006*
LVI (yes vs. no)	0.48 (0.31–0.76)	0.001*	0.63 (0.43–0.93)	0.019*
T stage (T1–2 vs. T3–4)	0.52 (0.33–0.83)	0.006*	0.55 (0.36–0.82)	0.003*
*N stage*		0.008*		–
N0 versus N3	0.28 (0.10–0.58)	0.001*	–	–
N1 versus N3	0.52 (0.26–1.02)	0.057	–	–
N2 versus N3	0.48 (0.22–1.03)	0.061	–	–
M stage (M0 vs. M1)	–	–	0.40 (0.18–0.90)	0.027*
*TNM stage*		< 0.001*		< 0.001*
IB versus IV	0.11 (0.04–0.29)	< 0.001*	0.19 (0.09–0.39)	< 0.001*
II versus IV	0.22 (0.16–0.42)	< 0.001*	0.30 (0.17–0.54)	< 0.001*
III versus IV	0.50 (0.28–0.89)	0.019*	0.64 (0.37–1.11)	0.111
CT cycle (< 4 vs. ≥ 4)	2.16 (1.39–3.37)	0.001*	2.32 (1.55–3.48)	< 0.001*

*SCEC* small cell esophageal carcinoma, *CSS* cancer-specific survival, *DFS* disease-free survival, *CT* chemotherapy, *NEC* neuroendocrine carcinoma, *ESCC* esophageal squamous cell carcinoma, *LVI* lymphovascular invasion, *HR* hazard ratio, *CI* confidence interval

**Fig. 4:**
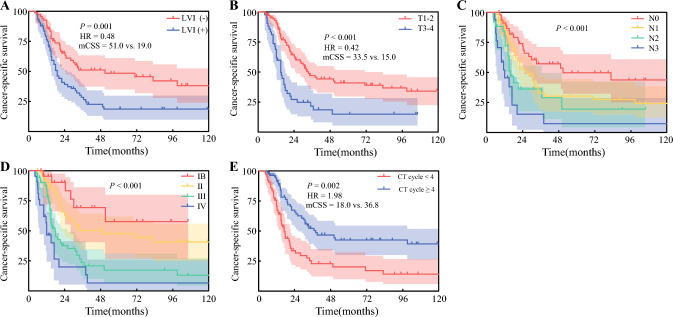
CSS curves stratified by (**a**) LVI; (**b**) T stage; (**c**) N stage; (**d**) TNM stage; and (**e**) CT cycle in SCEC patients. *CSS* cancer-free survival, *LVI* lymphovascular invasion, *TNM* tumor lymph node metastasis, *CT* chemotherapy, *SCEC* small cell esophageal carcinoma, *HR* hazard ratio, *mCSS* median cancer-specific survival

For CSS, univariate analyses associated LVI, histologic subtype, tumor length, T stage, N stage, and CT cycles with outcomes (ESM Table 5), while multivariate analysis identified LVI, advanced T/N/TNM stage, and < 4 CT cycles as independent predictors of reduced CSS (Fig. 4).

Notably, neither LNMs patterns nor NSM status correlated with DFS or CSS in SCEC patients.

## Discussion

An increasing number of studies have demonstrated that surgery-based multimodality treatment is one of the radical approaches for resectable SCEC; however, specific aspects such as the optimal extent of lymphadenectomy, indications for postoperative adjuvant therapy, and postoperative recurrence patterns remain understudied. Given the rarity of SCEC, establishing an optimal treatment paradigm through randomized controlled clinical trials remains unfeasible. This study systematically analyzes the clinicopathological features, lymphatic metastases, and postoperative recurrence patterns in resectable SCEC, aiming to enhance its understanding and provide actionable insights for refining clinical management strategies.

SCEC is a highly aggressive primary neuroendocrine carcinoma of the esophagus, exhibiting a strong propensity for high invasiveness and early lymph node or distant metastasis. Our data revealed that SCEC predominantly arises in the middle and lower esophagus, with no cases of carcinoma in situ and only two T1a lesions (1.4%) detected. When compared with the pattern of LNMs of ESCC reported in our prior study under standardized surgical procedures and balanced tumor stage, SCEC exhibited a numerically higher LNM rate (68.0 vs. 52.5%) and a significantly increased LNM incidence (11.3 vs. 7.9%), suggesting earlier lymphatic dissemination.^15^ Regarding LNM patterns, SCEC demonstrated concordance with established ESCC patterns: 59% of SCEC patients displayed proximal LNMs. Ut SCEC metastasized preferentially to the lymph nodes of Um, Mt SCEC exhibited diffuse lymph nodes spread with a bidirectional metastasis trend, and Lt SCEC showed a downward metastasis trend, primarily involving the Lm and abdomen lymph nodes. LNMs in the Um were common, regardless of the tumor location. Abdominal LNMs were mainly in the perigastric areas. The study found that LNM patterns related to the anatomic structure of the esophagus and the primary tumor site still existed. The No. 106rec lymph node is the most commonly metastasized Um lymph node, with metastatic rates of 75.0%, 24.7%, and 18% in the Ut, Mt, and Lt segments, respectively. Notably, SCEC demonstrates a propensity for abdominal LNM, with observed metastasis rates of 37.5, 28.1, and 48.0% in the Ut, Mt, and Lt, respectively, compared with the 10.3, 24.8, and 39.7% rates we previously documented for ESCC. Given the small Ut cohort in this study, this finding requires validation in larger multi-institutional studies. NSM demonstrates comparable prevalence in SCEC and ESCC,^15,19^ with our study revealing a 26.0% NSM incidence in SCEC. Similarly, these skip metastases predominantly exhibited caudal-directional patterning. These findings highlight the importance of comprehensive dissection of abdominal lymph nodes in SCEC surgeries due to its aggressive metastatic behavior.

Univariate regression analysis identified greater tumor invasion depth, longer tumor length, and LVI as risk factors for LNM in SCEC; however, multivariate analysis confirmed LVI as the sole independent risk factor. Our study revealed a significantly higher prevalence of LVI in SCEC versus ESCC (55.1% vs. 20.7–32.3%^20–23^). LVI refers to tumor cell invasion into blood or lymphatic vessels, which serve as pathways for the penetration of tumor cells.^20–23^ Previous studies have established an association between LVI and LNMs in ESCC.^24^ The elevated LVI rate in SCEC likely contributes to early LNMs and hematogenous spread, explaining its aggressive clinical behavior. Furthermore, tumor depth and length showed a significant correlation with LVI. Thus, we hypothesized that LVI, tumor invasion depth, and tumor length are all associated with LNM in SCEC; however, since LVI exhibited the strongest effect, the influence of tumor depth and length may have been overshadowed in multivariate analysis.

Regarding postoperative recurrence, distant metastases occurred in 77.2% of SCEC patients, whereas only 22.8% exhibited locoregional recurrence as the initial failure pattern. The most common distant metastatic sites were the liver and non-regional lymph nodes. Compared with ESCC, SCEC demonstrated a significantly higher tendency for distant metastasis as the first recurrence, emphasizing its aggressive metastatic potential and the unmet need for improved systemic therapies. Neither the use of ACT nor the number of CT cycles altered the recurrence patterns, with distant metastases remaining predominant. However, ≥ 4 ACT cycles might improve prognosis by reducing recurrence in a subset of patients. Notably, we observed regional LNMs as the first recurrence in 44.6% of SCEC patients, primarily in the No. 106rec and perigastric lymph nodes, underscoring the necessity of thorough lymphadenectomy in these regions.

The pathogenesis of SCEC remains poorly understood. Identifying prognostic determinants and improving survival rates represent critical research priorities. This study evaluated prognostic factors in resectable SCEC and revealed that LVI, advanced tumor stage, and fewer CT cycles were significantly associated with worse DFS and CSS, whereas histologic subtype correlated only with progression-free survival. Neither LNM patterns nor NSM exhibited significant associations with DFS or CSS. Under uniform lymphadenectomy criteria at our institution, SCEC did not show a significantly higher NSM incidence than ESCC. Prior studies have demonstrated no significant correlation between NSM and overall survival OS in ESCC,^25^ a finding confirmed in this study. NSM may not reflect inherently aggressive tumor biology or advanced disease stages; thus, if contiguous and skip LNMs receive equivalent treatment, NSM likely does not independently affect prognosis.

The TNM staging system for esophageal cancer, the paramount and widely recognized prognostic framework, acknowledges the impact of LVI on prognosis;^20,26^ however, due to the rarity of SCEC, no TNM system specific to this malignancy exists. Moreover, most prognostic factors identified in retrospective studies are clinical characteristics, with minimal exploration of biomarkers. Additionally, prior research lacks consensus on clinical prognostic indicators, and DFS-associated factors in SCEC remain underreported. This study highlights the significant influence of TNM stage (integrated with LNM) and LVI on DFS and CSS in SCEC patients. Current treatment strategies for SCEC lag far behind those for ESCC and small cell lung cancer, both of which have entered the era of immunotherapy with demonstrated survival benefits. Given the absence of evidence supporting targeted therapy or immunotherapy for SCEC, our analysis suggests that ≥ 4 CT cycles may improve outcomes, a finding supported by retrospective studies^5,8^ but requiring validation through higher-level evidence.

In summary, due to the early LNM of SCEC, high LVI prevalence, and substantial risk of distant metastatic recurrence, SCEC presents formidable therapeutic challenges and demonstrates dismal prognosis. This study constitutes the largest and most systematic investigation to date of LNM and recurrence patterns in SCEC, offering crucial evidence for optimizing treatment strategies and surgical approaches. However, several limitations must be acknowledged. First, as a single-center retrospective study, although surgical procedures were relatively standardized among surgeons, potential selection bias may exist. Moreover, the sample size remains limited due to the rarity of SCEC. Second, as reported in our prior study on LNM patterns in ESCC,^15^ not all patients received 3-FL lymphadenectomy, and routine pathological evaluation did not include detection of lymph node micrometastases. Additionally, perigastric lymph node classification lacked further anatomical refinement.

## Conclusions

The LNM patterns of SCEC adhere to the esophageal anatomical properties and tumor location. Compared with ESCC, SCEC demonstrates more extensive nodal dissemination and greater propensity for abdominal LNM. The elevated LVI incidence in SCEC emerges as the dominant predictor of nodal metastasis, surpassing tumor depth and longitudinal extension in significance. We strongly advocate thorough intraoperative dissection of No. 106rec and abdominal lymph nodes, supplemented with ≥ 4 cycles of ACT.

## Supplementary Information

Below is the link to the electronic supplementary material.

Supplementary file1 (DOCX 42 KB)

## Data Availability

The datasets used and/or analyzed during the current study are available from the corresponding author on reasonable request.

## References

[1] Bray F, Laversanne M, Sung H, Ferlay J, Siegel RL, Soerjomataram I, et al. Global cancer statistics 2022: GLOBOCAN estimates of incidence and mortality worldwide for 36 cancers in 185 countries. *CA Cancer J Clin*. 2024;74:229–63. 38572751 10.3322/caac.21834

[2] Lerut T, De Leyn P, Coosemans W, Van Raemdonck D, Scheys I, LeSaffre E. Surgical strategies in esophageal carcinoma with emphasis on radical lymphadenectomy. *Ann Surg*. 1992;216:583–90. 1444650 10.1097/00000658-199211000-00010 PMC1242677

[3] Greenstein AJ, Litle VR, Swanson SJ, Divino CM, Packer S, Wisnivesky JP. Prognostic significance of the number of lymph node metastases in esophageal cancer. *J Am Coll Surg*. 2008;206:239–46. 18222375 10.1016/j.jamcollsurg.2007.09.003

[4] Akiyama H, Tsurumaru M, Udagawa H, Kajiyama Y. Radical lymph node dissection for cancer of the thoracic esophagus. *Ann Surg*. 1994;220:364–72. 8092902 10.1097/00000658-199409000-00012 PMC1234394

[5] Yan H, Zhu H, Cai Y, Xin D, Cai G, Zou B, et al. Treatment strategies for limited-stage small cell carcinoma of the esophagus: evidence from a Chinese multicenter cohort study and the American SEER database. *J Thorac Dis*. 2024;16:7787–96. 39678850 10.21037/jtd-24-1394 PMC11635257

[6] Li R, Yang Z, Shao F, Cheng H, Wen Y, Sun S, et al. Multi-omics profiling of primary small cell carcinoma of the esophagus reveals RB1 disruption and additional molecular subtypes. *Nat Commun*. 2021;12:3785. 34145257 10.1038/s41467-021-24043-6 PMC8213753

[7] Wang F, Liu DB, Zhao Q, Chen G, Liu XM, Wang YN, et al. The genomic landscape of small cell carcinoma of the esophagus. *Cell Res*. 2018;28:771–4. 29728688 10.1038/s41422-018-0039-1 PMC6028429

[8] Zhu J, Wang Y, Sun H, Zhang Y, Zhang W, Shen W, et al. Surgery versus radiotherapy for limited-stage small cell esophageal carcinoma: A multicenter, retrospective, cohort study in China (ChiSCEC). *Int J Surg*. 2024;110:956–64. 37995095 10.1097/JS9.0000000000000912 PMC10871645

[9] Xu L, Yang YS, Li B, Cao YQ, Lin SY, Yu YK, et al. Multimodality therapy and survival outcomes in resectable primary small cell carcinoma of the esophagus: A multicenter retrospective study. *Ann Surg Oncol*. 2025;32:848–59. 39557721 10.1245/s10434-024-16532-x

[10] Xu L, Li Y, Liu X, Sun H, Zhang R, Zhang J, et al. Treatment strategies and prognostic factors of limited-stage primary small cell carcinoma of the esophagus. *J Thorac Oncol*. 2017;12:1834–44. 29024756 10.1016/j.jtho.2017.09.1966

[11] Situ D, Lin Y, Long H, Zhang L, Lin P, Zheng Y, et al. Surgical treatment for limited-stage primary small cell cancer of the esophagus. *Ann Thorac Surg*. 2013;95:1057–63. 23333059 10.1016/j.athoracsur.2012.11.014

[12] Meng MB, Zaorsky NG, Jiang C, Tian LJ, Wang HH, Liu CL, et al. Radiotherapy and chemotherapy are associated with improved outcomes over surgery and chemotherapy in the management of limited-stage small cell esophageal carcinoma. *Radiother Oncol*. 2013;106(3):317–22. 23498325 10.1016/j.radonc.2013.01.008

[13] Li J, Ma J, Wang H, Niu J, Zhou L. Population-based analysis of small cell carcinoma of the esophagus using the SEER database. *J Thorac Dis*. 2020;12:3529–38. 32802432 10.21037/jtd-20-1428 PMC7399392

[14] Jeene PM, Geijsen ED, Muijs CT, Rozema T, Aleman BMP, Muller K, et al. Small cell carcinoma of the esophagus: A nationwide analysis of treatment and outcome at patient level in locoregional disease. *Am J Clin Oncol*. 2019;42:534–8. 31021827 10.1097/COC.0000000000000546 PMC6554014

[15] Li B, Chen H, Xiang J, Zhang Y, Li C, Hu H, et al. Pattern of lymphatic spread in thoracic esophageal squamous cell carcinoma: a single-institution experience. *J Thorac Cardiovasc Surg*. 2012;144:778–85. 22889480 10.1016/j.jtcvs.2012.07.002

[16] Li H, Zhang Y, Cai H, Xiang J. Pattern of lymph node metastases in patients with squamous cell carcinoma of the thoracic esophagus who underwent three-field lymphadenectomy. *Eur Surg Res*. 2007;39:1–6. 17106199 10.1159/000096925

[17] Rice TW, Ishwaran H, Ferguson MK, Blackstone EH, Goldstraw P. Cancer of the esophagus and esophagogastric junction: an eighth edition staging primer. *J Thorac Oncol*. 2017;12:36–42. 27810391 10.1016/j.jtho.2016.10.016 PMC5591443

[18] Japanese Society for Esophageal Diseases. Guide lines for the clinical and pathologic studies on carcinoma of the esophagus. *Jpn J Surg*. 1976;6:69–78. 994356 10.1007/BF02468889

[19] Zhu Z, Yu W, Li H, Zhao K, Zhao W, Zhang Y, et al. Nodal skip metastasis is not a predictor of survival in thoracic esophageal squamous cell carcinoma. *Ann Surg Oncol*. 2013;20:3052–8. 23686016 10.1245/s10434-013-2987-5

[20] Zhou J, Yang Y, Zhang H, Luan S, Xiao X, Li X, et al. Lymphovascular and perineural invasion after neoadjuvant therapy in esophageal squamous carcinoma. *Ann Thorac Surg*. 2023;115:1386–94. 36027933 10.1016/j.athoracsur.2022.07.052

[21] Wang S, Chen X, Fan J, Lu L. Prognostic significance of lymphovascular invasion for thoracic esophageal squamous cell carcinoma. *Ann Surg Oncol*. 2016;23:4101–9. 27436201 10.1245/s10434-016-5416-8

[22] Tang B, Wu F, Peng L, Leng X, Han Y, Wang Q, et al. Computed tomography-based radiomics nomogram for prediction of lympho-vascular and perineural invasion in esophageal squamous cell cancer patients: a retrospective cohort study. *Cancer Imaging*. 2024;24:131. 39367492 10.1186/s40644-024-00781-w PMC11451056

[23] Zhang L, Shao J, Liu Z, Pan J, Li B, Yang Y, et al. Occurrence and prognostic value of perineural invasion in esophageal squamous cell cancer: a retrospective study. *Ann Surg Oncol*. 2022;29:586–97. 34426885 10.1245/s10434-021-10665-z

[24] Yang J, Lu Z, Li L, Li Y, Tan Y, Zhang D, et al. Relationship of lymphovascular invasion with lymph node metastasis and prognosis in superficial esophageal carcinoma: systematic review and meta-analysis. *BMC Cancer*. 2020;20:176. 32131772 10.1186/s12885-020-6656-3 PMC7057611

[25] Gu-Ha AL, Xu ZJ, Yao P, Zhong X, Wang YC, Lin YD. Prognostic value of node skip metastasis on esophageal cancer: a systematic review and meta-analysis. *Asian J Surg*. 2022;45:2601–7. 35221181 10.1016/j.asjsur.2021.12.071

[26] Rice TW, Patil DT, Blackstone EH. 8th edition AJCC/UICC staging of cancers of the esophagus and esophagogastric junction: application to clinical practice. *Ann Cardiothorac Surg*. 2017;6:119–30. 28447000 10.21037/acs.2017.03.14 PMC5387145

