# Supplementary material for: Pattern of Lymph Node Metastases and Recurrence in Thoracic Small Cell Esophageal Carcinoma: A Single-Institution Experience
Source: Ann Surg Oncol. 2025 Oct 7;33(2):871–80. doi: 10.1245/s10434-025-18425-z (PMC12765737; doi:10.1245/s10434-025-18425-z)
Supplement: Supplementary file 1 — Supplementary file1 (DOCX 42 KB) [file 10434_2025_18425_MOESM1_ESM.docx]

**SUPPLEMENTAL INFORMATION**

**Supplemental TABLE 1** The frequency of lymphovascular invasion in SCEC

| **Variables** | **Lymphovascular invasion** | | ***P* value** |
| --- | --- | --- | --- |
|  | Negative (%) | Positive (%) |  |
| **T stage** |  |  | 0.010* |
| T1a | 1(50) | 1(50) |  |
| T1b | 30(61.2) | 19(38.8) |  |
| T2 | 23(42.6) | 31(57.4) |  |
| T3 | 12(32.4) | 25(67.6) |  |
| T4a | 0(0.0) | 5(100.0) |  |
| **N stage** |  |  | <0.001* |
| N0 | 36(50) | 11(23.4) |  |
| N1 | 20(35.1) | 37(64.9) |  |
| N2 | 10(38.5) | 16(61.5) |  |
| N3 | 0(0.0) | 17(100.0) |  |
| **Tumor length** |  |  | 0.003* |
| ≤3cm | 37(56.1) | 29(43.9) |  |
| 3-5cm | 27(42.2) | 37(57.8) |  |
| >5cm | 2(11.8) | 15(88.2) |  |

*SCEC* small cell esophageal carcinoma.

**Supplemental TABLE 2 Univariate analysis of factors associated with LVI**

| **Variables** | **OR** | **95%CI** | ***P* value** |
| --- | --- | --- | --- |
| **Length of disease (cm)** |  |  | 0.011* |
| ≤3 *vs.* >5 | 0.105 | 0.022-0.494 | 0.004* |
| 3-5 *vs.* >5 | 0.183 | 0.039-0.876 | 0.032* |
| **T stage** (T1-2 *vs.* T3-4) | 0.378 | 0.175-0.817 | 0.013* |
| **LNM** (no *vs.* yes) | 0.131 | 0.059-0.291 | <0.001* |

*LVI* lymphovascular invasion; *OR* odds ratio; *CI* confidence interval; *LNM* lymph node metastasis.

**Supplemental TABLE 3 Metastatic sites after first line treatment in SCEC patients**

| **Metastatic site** | ***N*** |
| --- | --- |
| Liver metastasis | 29 |
| Lung metastasis | 14 |
| Bone metastasis | 12 |
| Brain metastasis | 10 |
| Peritoneal metastasis | 3 |
| Pleural metastasis | 2 |
| Pancreatic metastasis | 1 |
| Subcutaneous metastasis | 1 |
| Distant LNM | 29 |
| Supraclavicular lymph node | 12 |
| Retroperitoneal lymph node | 9 |
| Cervical lymph node | 7 |
| Axillary lymph node | 2 |

*SCEC* small cell esophageal carcinoma; *LNM* lymph node metastasis.

**Supplemental TABLE 4** Univariate analysis of the prognostic factors for CSS in SCEC patients

| **Variables** | **N(%)** | **Median CSS (m, 95%CI)** | **Chi-square** | ***P* value** | **HR(95%CI)** | ***P* value** |
| --- | --- | --- | --- | --- | --- | --- |
| **Pathology** |  |  | 8.5 | 0.014* |  | 0.023* |
| Pure SCEC | 115(78.2) | 22.0(16.7-27.3) |  |  | 0.27(0.06-1.10) | 0.067 |
| Mixed ESCC/EAC | 30(20.4) | 49.4(28.1-70.7) |  |  | 0.15(0.03-0.67) | 0.013* |
| Mixed large cell NEC | 2(1.4) | 6.2(-) |  |  | 1.00 |  |
| **LVI** |  |  | 10.8 | 0.001* |  |  |
| Yes | 81(55.1) | 19.0(15.2-22.8) |  |  | 1.00 |  |
| No | 66(44.9) | 51.0(0.0-103.9) |  |  | 0.48(0.31-0.76) | 0.001* |
| **CT cycle** |  |  | 9.8 | 0.002* |  |  |
| <4 | 72(49.0) | 18.0(13.7-22.3) |  |  | 1.98(1.28-3.06) | 0.002* |
| ≥4 | 75(51.0) | 36.8(27.8-45.8) |  |  | 1.00 |  |
| **Length of disease** |  |  | 6.6 | 0.037* |  | 0.043* |
| ≤3cm | 66(44.9) | 31.0(21.8-40.2) |  |  | 0.44(0.23-0.85) | 0.014* |
| 3-5cm | 64(43.5) | 21.1(16.1-26.1) |  |  | 0.61(0.32-1.17) | 0.138 |
| >5cm | 17(11.6) | 15.0(6.4-23.6) |  |  | 1.00 |  |
| **T stage** |  |  | 16.3 | <0.001* |  |  |
| T1-2 | 105(71.4) | 33.5(24.9-42.1) |  |  | 0.42(0.27-0.65) | <0.001* |
| T3-4 | 42(28.6) | 15.0(11.3-18.7) |  |  | 1.00 |  |
| **N stage** |  |  | 23.7 | <0.001* |  | <0.001* |
| N0 | 47(32.0) | 51.0(0.0-110.8) |  |  | 0.21(0.11-0.42) | <0.001* |
| N1 | 57(38.8) | 28.6(19.6-37.6) |  |  | 0.36(0.19-0.67) | 0.001* |
| N2 | 26(17.7) | 16.0(11.6-20.4) |  |  | 0.52(0.26-1.05) | 0.070 |
| N3 | 17(11.6) | 12.0(6.3-17.7) |  |  | 1.00 |  |
| **TNM stage** |  |  | 34.8 | <0.001* |  | <0.001* |
| IB | 23(15.6) | - |  |  | 0.14(0.05-0.35) | <0.001* |
| II | 48(32.7) | 49.4(0.0-111.1) |  |  | 0.23(0.12-0.44) | <0.001* |
| III | 55(37.4) | 17.9(13.3-22.5) |  |  | 0.56(0.32-1.00) | 0.048* |
| IV | 21(14.3) | 12.0(7.5-16.5) |  |  | 1.00 |  |

*SCEC* small cell esophageal carcinoma; *CSS* cancer-specific survival; *HR* hazard ratio; *CI* confidence interval; *CT* chemotherapy; *NEC* neuroendocrine carcinoma; *ESCC* esophageal squamous cell carcinoma; *EAC* esophageal adenocarcinoma; *LVI* lymphovascular invasion.

**Supplemental TABLE 5** Univariate analysis of the prognostic factors for DFS in SCEC patients

| **Variables** | **N(%)** | **Median DFS (m，95%CI)** | **Chi-square** | ***P* value** | **HR(95%CI)** | ***P* value** |
| --- | --- | --- | --- | --- | --- | --- |
| **Pathology** |  |  | 11.4 | 0.003* |  | 0.009* |
| Pure SCEC | 115(78.2) | 12.0(0.4-13.6) |  |  | 0.18(0.04-0.76) | 0.020* |
| Mixed ESCC /EAC | 30(20.4) | 17.3(14.3-20.3) |  |  | 0.11(0.02-0.49) | 0.004* |
| Mixed large cell NEC | 2(1.4) | 5.3(-) |  |  | 1.00 |  |
| **LVI** |  |  | 5.5 | 0.019* |  |  |
| Yes | 81(55.1) | 11.2(9.8-12.6) |  |  | 1.00 |  |
| No | 66(44.9) | 15.0(11.2-18.8) |  |  | 0.64(0.43-0.94) | 0.021* |
| **ACT** |  |  | 10.3 | 0.001* |  |  |
| Yes | 118(80.3) | 13.4(11.1-15.7) |  |  | 0.49(0.32-0.75) | 0.001* |
| No | 29(19.7) | 6.9(3.7-10.1) |  |  | 1.00 |  |
| **CT cycle** |  |  | 16.1 | <0.001* |  |  |
| < 4 | 72(49.0) | 10.0(8.2-11.8) |  |  | 2.16(1.46-3.18) | <0.001* |
| ≥ 4 | 75(51.0) | 15.2(10.9-19.5) |  |  | 1.00 |  |
| **T stage** |  |  | 10.0 | 0.002* |  |  |
| T1-2 | 105(71.4) | 14.0(10.9-17.1) |  |  | 0.53(0.36-0.79) | 0.002* |
| T3-4 | 42(28.6) | 8.3(5.5-11.1) |  |  | 1.00 |  |
| **N stage** |  |  | 15.9 | 0.001* |  | 0.002* |
| N0 | 47(32.0) | 17.0(12.5-21.5) |  |  | 0.34(0.18-0.63) | 0.001* |
| N1 | 57(38.8) | 13.3(9.9-16.7) |  |  | 0.49(0.27-0.88) | 0.017* |
| N2 | 26(17.7) | 9.5(6.6-12.4) |  |  | 0.75(0.39-1.45) | 0.391 |
| N3 | 17(11.6) | 7.8(2.1-13.5) |  |  | 1.00 |  |
| **M stage** |  |  | 6.1 | 0.013* |  |  |
| M0 | 139(94.6) | 13.0(11.2-14.8) |  |  | 0.39(0.18-0.85) | 0.017* |
| M1 | 8(5.4) | 4.0(0.0-8.2) |  |  | 1.00 |  |
| **TNM stage** |  |  | 27.9 | <0.001* |  | <0.001* |
| IB | 23(15.6) | 18.0(16.7-19.3) |  |  | 0.25(0.12-0.51) | <0.001* |
| II | 48(32.7) | 17.0(11.9-22.1) |  |  | 0.29(0.16-0.53) | <0.001* |
| III | 55(37.4) | 11.0(8.1-13.9) |  |  | 0.64(0.37-1.09) | 0.098 |
| IV | 21(14.3) | 7.8(4.8-10.8) |  |  | 1.00 |  |

*SCEC* small cell esophageal carcinoma; *DFS* disease-free survival; *HR* hazard ratio; *CI* confidence interval; *CT* chemotherapy; *ACT* adjuvant chemotherapy; *NEC* neuroendocrine carcinoma; *ESCC* esophageal squamous cell carcinoma; *EAC* esophageal adenocarcinoma; *LVI* lymphovascular invasion.
